# The Tyrolean early vascular ageing-study (EVA-Tyrol): study protocol for a non-randomized controlled trial: Effect of a cardiovascular health promotion program in youth, a prospective cohort study

**DOI:** 10.1186/s12872-020-01357-9

**Published:** 2020-02-05

**Authors:** Benoît Bernar, Nina Gande, Katharina A. Stock, Anna Staudt, Raimund Pechlaner, Ralf Geiger, Andrea Griesmacher, Stefan Kiechl, Michael Knoflach, Ursula Kiechl-Kohlendorfer

**Affiliations:** 1 Department of Neurology, Medical University of Innsbruck, Anichstrasse 35, A-6020 Innsbruck, Austria; 2 Department of Pediatrics II, Medical University of Innsbruck, Anichstrasse 35, A-6020 Innsbruck, Austria; 3 Department of Pediatrics III, Medical University of Innsbruck, Anichstrasse 35, A-6020 Innsbruck, Austria; 4 Department of Pediatrics, Bruneck Hospital, Bruneck, Italy; 5 Central Institute of Clinical Chemistry and Laboratory Medicine (ZIMCL), Medical University of Innsbruck, Anichstrasse 35, A-6020 Innsbruck, Austria

## Abstract

**Background:**

According to the World Health Organization, cardiovascular diseases (CVDs) are the leading non-communicable cause of death. Awareness of the individual risk profile is crucial to implement a healthy lifestyle and prevent CVDs. Multiple studies demonstrated that atherosclerosis, the main cause of CVDs, begins early in life. Therefore, it may be necessary to start prevention programs already in childhood.

**Methods:**

The EVA-Tyrol study is a population-based non-randomized controlled trial that will prospectively enroll 2000 participants from high schools and training companies in North- and East-Tyrol (Austria) and South-Tyrol (Italy). Participants will be assigned to either an intervention (*n* = 1500) or a control (*n* = 500) group. Intervention group participants will be enrolled at the 10th school grade (mean age 15–16 years), undergo two examinations within a two-year interval, with follow-up at the 12th grade (mean ages 17–18 years). Control group participants will be enrolled at the 12th grade (mean age 17–18 years). Medical examination will include anthropometric measurements, comprehensive lifestyle and dietary questionnaires, a fasting blood sample, high-resolution ultrasound of the carotid arteries, and measurement of carotid-femoral pulse wave velocity. Active intervention will consist of (1) enhancing knowledge about CVDs, (2) individual medical counseling based on the results of the baseline examination, (3) an online health promotion tool and (4) involvement of participants in planning and implementation of health promotion projects. Effectiveness of the intervention will be assessed by comparing the proportion subjects with ideal health metrics as defined by the American Heart Association between study groups.

**Discussion:**

This study aims to improve cardiovascular health in Tyrolean adolescents by demonstrating the efficacy of a multi-layer health promotion program and may yield novel insights into the prevalence of vascular risk conditions and mechanisms of early vascular pathologies in adolescents.

**Trial registration:**

EVA-Tyrol has been retrospectively registered at clinicaltrials.gov under NCT03929692 since April 29, 2019.

## Background

According to the world health organization (WHO), 17.7 million people died from CVDs in 2015 [1, 2], with ischemic heart disease and stroke accounting for 26.6% of all deaths worldwide [3]. The Committee on Preventing the Global Epidemic of Cardiovascular Disease recommended that apart from early diagnosis and management of CVDs interventions at ‘all stages of life course’ should be performed in order to ‘promote cardiovascular health by preventing acquisition and augmentation of risk’ [4]. Awareness of the individual risk profile and subsequent modification of risk factors and risk behavior are prerequisites for effective prevention. However, it is unclear at which age best to initiate prevention.

While CVDs occur predominately in the elderly, atherosclerosis starts in early life and might even be influenced by fetal and postnatal development. According to the Barker hypothesis, early life factors such as being born preterm or small for gestational age, or impaired fetal growth or inadequate weight gain in the first years of life may predispose to CVDs later in life [5–8]. Histologic necropsy studies found coronary atherosclerotic plaques in 12% of adolescents and 28% of young adults [9]. Classical cardiovascular risk factors like smoking, physical inactivity and unhealthy diet are present not only in adults and adolescents but also in the pediatric population [10–12]. In addition, unfavorable health behaviors like sedentary lifestyle, smoking and alcohol consumption may be acquired in late childhood [13–15] and frequently persist in adulthood. Cohort studies have demonstrated the effects of risk factors on early atherosclerotic vessel wall thickening using high-resolution ultrasonography in children, adolescents and young adults [16, 17]. Conversely, a more favorable cardiovascular risk profile in childhood (as defined by the AHA) is associated with a lower aortic intima-media thickness (IMT) and a better aortic elasticity [18], and with a reduced risk of hypertension, metabolic-syndrome and elevated low-density lipoprotein cholesterol in adulthood [19]. Early correction of an unfavorable lifestyle can prevent CVDs in later life [20]. Adverse effects of obesity may be reversed by early weight reduction [20]. In Children, lifestyle and dietary counseling exhibits favorable effects on their cardiovascular risk profile without harmful side-effects [18, 21].

In summary, it is well established that cardiovascular risk factors (CVRFs) are related to early vascular ageing and early atherosclerotic wall changes in children and young adults. Promotion of a healthy lifestyle and control of CVRF. In youth presents the opportunity to reverse these changes and prevent persistence of risk conditions into adulthood. The current study will evaluate the efficacy of a defined cardiovascular health promotion program in facilitating risk factor control.

### Trial aim and objectives

The aim of EVA-Tyrol is to promote cardiovascular health in high schools/training companies by an interventional health promotion program and to acquire data on its efficacy in a cohort of Tyrolean adolescents with a mean age of 15–16 years.

The primary objectives of EVA-Tyrol are:

- To assess health in the Tyrolean youth
- To survey the effect of a multi-layer health promotion program in this age group
- To study the effects of neonatal and childhood weight gain on CVRF in youth
- To assess the effects of being born preterm or small for gestational age on vascular health in youth
- To determine the effects of CVRF on vascular health in youth
- To explore the effects of lifestyle on vascular health in youth
- To establish a serum biobank

## Methods and design

### Study design

The EVA-study is a non-randomized controlled trial. Two thousand participants will be recruited from high schools and training companies spread over North- and East-Tyrol (Austria) and South-Tyrol (Italy). Study participants will be assigned to i) health intervention group (*n* = 1500) or ii) control group (*n* = 500). For participants included in the intervention group two examinations will be scheduled within a two-year interval between the examinations. Participants will be in the 10th grade (mean age, 15–16 years) at the baseline and in the 12th grade (mean age, 17–18 years) at the follow-up examination. Between the examinations the health intervention program will be offered.

**Fig. 1:**
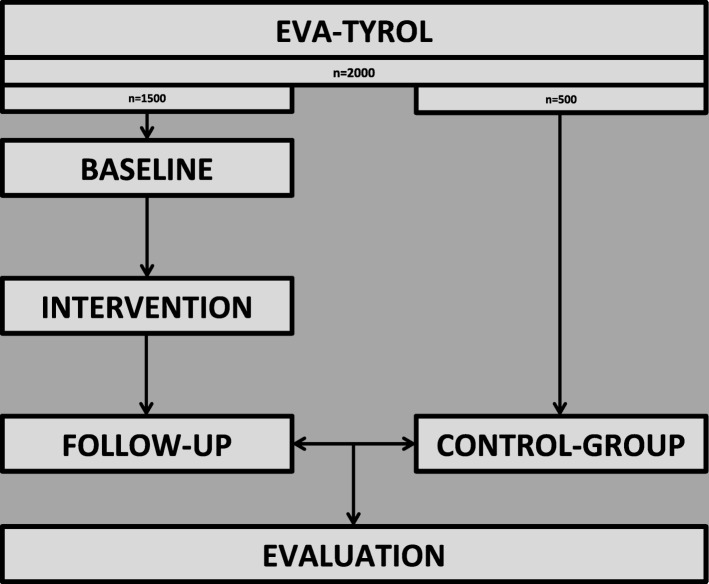
Schematic illustration of study design

Five hundred participants with a mean age of 17–18 years without participation in a health promotion program will serve as a control group. The prevalence of AHA health metrics in the intervention group will be compared to the prevalence in the control group (Fig. 1).

In addition to health metrics, EVA-Tyrol will evaluate further components and determinants of cardiovascular health of Tyrolean adolescents. In addition, we will collect information on the first 6 years of life including pre-, peri- and postnatal data prospectively documented in the mother-child booklet, the official Austrian pregnancy and early childhood medical record book.

### Participants

#### Recruitment

The local education authority (Landesschulrat for Tyrol) will brief all Tyrolean high schools about the project. Subsequently, the schools will be able to apply for study-inclusion. In order to ensure a homogenous regional and social distribution, principals of selected schools will be actively encouraged to participate. Schools and training companies will not receive financial compensation for participation. The two study centers will be Innsbruck (Austria) for North and East Tyrol and Bruneck (Italy) for South Tyrol. Moreover, Tyrolean training companies will be invited to participate. For participating schools and companies a project presentation on the background of CVD and concept of CVRF will be offered. Pupils and trainees will be briefed about the study procedure. In addition, a video summarizing study aims and procedures will be shown.

#### Inclusion criteria

Participants in the 10th to 12th grade (mean ages 15–16 at baseline for the intervention group and 17–18 years for the control group) will be enrolled. Informed consent by the participant and, for participants younger than 18 years, their legal representative will be obtained. Consent for inclusion of data from the “mother-child booklet” additional maternal informed consent will be obtained.

Schools and training companies will be randomly assigned to either the intervention or the control group. The examination of the intervention group will start first. Schools will be assigned to either group according to their available schedule. The exact number of participating schools/training companies will depend on the number of participants per school/company.

#### Exclusion criteria

None; previous or concurrent diseases are not an exclusion criterion.

### Data acquisition

**Fig. 2:**
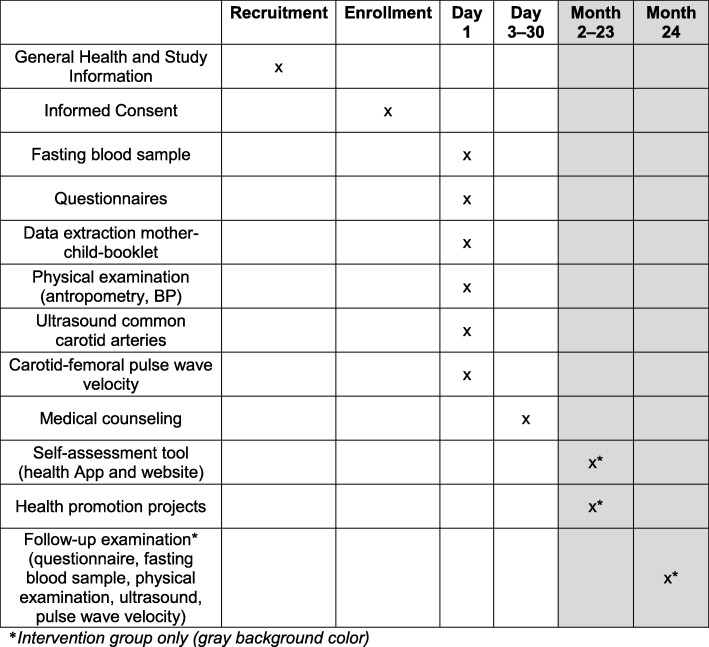
SPIRIT diagram: Timeframe of EVA-Tyrol

The study will take place at the schools’ or training companies’ sites. An overview of the trial procedures is depicted in Fig. 2. Data will be collected by a paper case report form (CRF) and include a self-administered and assisted questionnaire, a structured interview and a series of examinations (blood sampling, high-resolution ultrasound of the carotid arteries, tonometric measurement of carotid-femoral pulse-wave velocity, blood-pressure measurement and anthropometry). Data acquisition will be done by medical doctors assisted by medical students.

#### Questionnaire

Table 1 shows the structure of the employed CRF. The CRF is adapted from the Bruneck- [27], ARMY- [16], ARFY- [17] and HBSC (Health Behavior in School Children Study)- [22] studies and organized in three parts: the first part of the CRF is self-administered, the second part has to be completed under group supervision of an MD. The third part is a structured face-to-face questionnaire by an MD about the study’s main outcome parameters and previous medical history of each participant (Table 1). The family history for diabetes, hypertension or CVDs, is explored separately for each condition and rated as present when at least one first or 2 s degree relatives became diseased at an early age (women < 65 years, men < 55 years).

**Table 1:** Overview over the EVA-Tyrol questionnaire

Self-administered		
Overall health and life satisfaction, lifestyle factors, social background	21 items	Health Behaviour in School Children -Study [22]
Food habits	23 items	The Adolescent Food Habits Checklist [23]
Nutritional knowledge	16 items	Turconi score Section E, G and H [24]
Participation and perception of health promotion	12 items	
Assisted		
Baecke score for physical activity	20 items	[25]
Score for Allergic Rhinitis	9 items	[26]
Traffic-Exposure	3 items	Taken from the ARMY [16] and ARFY [17] studies
Food frequency questionnaire	90 items	Taken from the ARMY [16], ARFY [17] and Bruneck [27] studies
Face-to-face interview		
Dietary interview	7 items	According to the AHA health metrics for youth [11, 28]
Physical activity	1 item	Moderate- and vigorous-intensity activity (minutes per day), AHA health metrics for youth [11, 28]
Smoking and alcohol consumption	12 items	Adapted from the Bruneck Study [27, 29]
Classical cardiovascular risk-factors and previous diseases as well as chronic infections.	17 items	Structured interview for known hypercholesterolemia, diabetes, hypertonia, diseases of the heart, vasculature, thyroid glands, liver, lung, known neoplasias, chronic infections of lung, sinuses, urinary tract, skin or chronic dental infections.
Family history for CVD	3 items	Premature CVDs (women < 65 years, men < 55 years), hypertension or diabetes in one 1st or two 2nd degree relatives.
Allergies and atopic predisposition	11 items	Structured interview for history of allergies, clinical symptoms and medical therapy.
Headache history	8 items	Classification of the international headache society (ICHD-3) [30]
Medication use	9 items	Structured interview for previous and current medication use

Table 1 shows an overview of the three parts (self-administered, assisted and face-to-face) of the EVA-Tyrol questionnaire. Left column – topic, middle column – number of questions, right column – source/description of questionnaire

#### Mother-child booklet data

In 1974 the Austrian government introduced a mother-child booklet (“Mutter-Kind Pass”) aiming to improve the health of pregnant women and children [31]. It consists of predefined examinations of the mother, the fetus, and the newborn and extends until the age of 6 years. Results of these examinations are documented in a booklet. As the child’s continuous financial governmental support is dependent on the completion of all examinations, adherence to the suggested medical examinations is high. Data extracted from the mother-child booklet is shown in Table 2. The Italian mother-child booklet is similar in content except for minor differences in examination intervals.

**Table 2:** Data extracted from the mother-child-booklet

Mother-child-booklet	
Maternal characteristics	Age at pregnancy Singleton/multiple pregnancy Body weight and length (begin and end of pregnancy) Blood pressure (begin and end of pregnancy) Smoking status (begin and end of pregnancy) Pre-existing conditions (diabetes, hypertension)
Pregnancy complications (yes/no)	Pathological oral glucose tolerance test Preeclampsia Hypertension Proteinuria
Characteristics of neonate	Date of birth Gestational age Apgar Score Umbilical blood pH Mode of birth Body weight Body length Head circumference
Data of the child of 8 follow-up examinations at week 4–7, month 3–5, 7–9, 10–14, 22–26, 34–38, 46–50 and 58–62	Date of examination Body weight Body length Head circumference

Table 2 shows an overview of the information extracted from the mother-child-booklet

#### Physical examination

Physical examination will be performed by study personnel. Anthropometric measurements will include size and weight as well as waist- and hip-circumference. Blood pressure will be measured three times in the sitting position after 5 min of rest by a standard digital haemodynamometer (Omron; Omron Healthcare, Lake Forest, Illinois, US) on both arms. High-resolution ultrasound of the carotid arteries will be performed with a portable GE medical Vivid_i_ ultrasound with a linear transducer 12 L RS probe (both General Electric, GE-Healthcare, Chicago, US). The intima-media thickness (IMT) will be assessed by experienced sonographers from the anterior and posterolateral view. Representative images will be stored digitally and IMT will be measured offline on the stored images on three representative locations in the distal proportion of the common carotid artery of both sides. Carotid-femoral pulse wave velocity and central blood pressure will be calculated as a surrogate for aortic stiffness from simultaneous recording of ten consecutive pulse waves of artefact-free cardiac cycles by applanation tonometry according to the manufacturer’s instructions (Complior-Analyze®, ALAMmedical, Paris, France).

#### Sample collection and analysis

Blood samples will be drawn after an overnight fastand will immediately be stored in cooling boxes at approximately 4 °C before direct transfer to the ISO-certified Central Institute for Medical and Chemical Laboratory Diagnostics of Innsbruck University Hospital. Parameters and measurement methods are detailed in Table 3. Long-term storage of serum and plasma samples will be at − 80 °C. The serum biobank will consist of serum and lithium-heparin plasma, as well as full blood for DNA extraction only for subjects that signed an additional informed consent form.

**Table 3:** ‘Routine lab parameters and methodology'. HPLC High pressure liquid chromatography, ECLIA Electrochemiluminescenceimmunoassay

Parameter	Unit	Method	Reagent	Analyzer
Glucose	mg/dl	Hexokinase method	Roche	Cobas 8000
HbA1c (DCCT/NGSP)	%	HPLC	Menarini	HA 8180 T
HbA1c (IFCC)	mmol/mol	HPLC	Menarini	HA 8180 T
Insulin	mU/l	ECLIA	Roche	Cobas 8000
Cholesterol	mg/dl	Enzymatic color assay	Roche	Cobas 8000
HDL-Cholesterol	mg/dl	Enzymatic color assay	Roche	Cobas 8000
LDL-Cholesterol	mg/dl	Enzymatic color assay	Roche	Cobas 8000
Triglyceride	mg/dl	Enzymatic color assay	Roche	Cobas 8000
Lipoprotein (a)	nmol/l	Particle-enhanced immunological clouding assay	Roche	Cobas 8000
Total-Homocystein	umol/l	Chemiluminescence microparticle immunoassay	Abbott	Architect
Urea	mg/dl	Kinetic test with urease and Glutamate dehydrogenase	Roche	Cobas 8000
Creatinine (enzym.-IDMS)	mg/dl	Enzymatic color assay	Roche	Cobas 8000
Total-protein	g/dl	Biuret test	Roche	Cobas 8000
Uric acid	mg/dl	Enzymatic color assay with uricase	Roche	Cobas 8000
Potassium	mmol/l	Indirect potentiometry	Roche	Cobas 8000
Calcium	mmol/l	Photometric with 5-Nitro-5′-methyl-BAPTA	Roche	Cobas 8000
GOT (ASAT)	U/l	According to IFCC recommendations, though optimized	Roche	Cobas 8000
GPT (ALAT)	U/l	According to IFCC recommendations, though optimized	Roche	Cobas 8000
Gamma-GT	U/l	Enzymatic color assay	Roche	Cobas 8000
Creatine kinase	U/l	Enzymatic UV-Assay	Roche	Cobas 8000
C-reactive protein	mg/dl	Particle-enhanced immunological clouding assay	Roche	Cobas 8000
Ferritin	ug/l	Particle-enhanced immunological clouding assay	Roche	Cobas 8000
TSH	mU/l	ECLIA	Roche	Cobas 8000
Free thyroxine (FT4)	pmol/l	ECLIA	Roche	Cobas 8000
Thyroglobulin	ug/l	ECLIA	Roche	Cobas 8000
Thyroglobulin-Antibodies	kU/l	ECLIA	Roche	Cobas 8000
ESR after 1 h	mm/h	Westergren-Method	Mechatronics	Starrsed Auto Compact
Leukocytes	G/l	Biofluorescence/flow cytometry	Sysmex	XE-5000
Absolute number of Neutrophiles	G/l	Biofluorescence/flow cytometry	Sysmex	XE-5000
Erythrocytes	T/l	Impedance method	Sysmex	XE-5000
Haemoglobin	g/l	Photocolorimetric	Sysmex	XE-5000
Hematocrit	l/l	Impedance method	Sysmex	XE-5000
Platelets	G/l	Impedance method	Sysmex	XE-5000
MCH	pg	Calculated	Sysmex	XE-5000
MCHC	g/l	Calculated	Sysmex	XE-5000
MCV	fl	Calculated	Sysmex	XE-5000
Erythrocyte-distributional width	%	Calculated	Sysmex	XE-5000
Band neutrophiles	%	Manual microscopy	manual	Light microscopy
Segmented neutrophiles	%	Biofluorescence/flow cytometry and light microscopy	Sysmex	XE-5000
Lymphocytes	%	Biofluorescence/flow cytometry and light microscopy	Sysmex	XE-5000
Lymphocytes	G/l	Biofluorescence/flow cytometry and light microscopy	Sysmex	XE-5000
Monocytes	%	Biofluorescence/flow cytometry and light microscopy	Sysmex	XE-5000
Eosinophils	%	Biofluorescence/flow cytometry and light microscopy	Sysmex	XE-5000
Basophils	%	Biofluorescence/flow cytometry and light microscopy	Sysmex	XE-5000
Plasma cells	%	Manual microscopy	Manual	Light microscopy
Folic acid	μg/l	Competitive binding assay	Roche	Cobas 8000
Vitamin B12	pmol/l	ECLIA	Roche	Cobas 8000

#### Cardiovascular health evaluation

As successfully applied in previous studies [18, 21], healthy lifestyle will be measured by the AHA’s seven health metrics (adapted by Lloyd-Jones [11] and Daniels [28]). Every health metric except smoking is categorized into ideal, moderate and poor health as defined in Table 4. Healthy diet is defined by the AHA based on expert opinion and on the dietary approach to stop hypertension (DASH) score. One point is scored for each of the following components: consumption of at least four portions of vegetables and fruits per day, at least three portions of fiber-rich nutriments per day, of fish at least two-times a week, a salt-free or salt-poor diet (less than 1.5 g/day) and not more than 1 Liter of sugar-rich drinks per week (max. 450 kcal/week) [11].

**Table 4:** ‘Seven AHA health metrics’

Health behavior goal:	Poor	Moderate	Ideal health
Smoking-habits	Smoked in the last 30 days	–	Never smoked, never smoked a whole cigarette
Body-Mass-Index	>95th percentile	85-95th percentile	<85th percentile
Physical activity	None	< 60 min moderate or intensive physical activity per day	≥ 60 min moderate or intensive physical activity per day
Healthy diet	0–1 components	2–3 components	4–5 components
Total-cholesterol	≥200 mg/dL	170–199 mg/dL	< 170 mg/dL
Blood pressure	>95th percentile	90-95th percentile or ≥ 120 systolic or ≥ 80 diastolic	<90th percentile
Fasting blood sugar	≥126 mg/dL	100–125 mg/dL	< 100 mg/dL

Table 4 contains the definition of poor, intermediate and ideal cardiovascular health according to the seven AHA health metrics adapted from [11] and [28]

### Intervention

Our health promotion program is based on (1) enhancing the knowledge about CVDs and related risk factors, (2) medical counseling and discussion of individual risk conditions and lifestyle, (3) providing a self-assessment tool to control and visualize chances in CVD risk conditions and (4) involvement of participants in the planning of health promotion projects.

Our health-promotion program will combine the running health-promotion programs “Gesunde Schule (healthy school)” and “Do-it-yourself!” of the Tyrolean regional medical insurance company (Tiroler Gebietskrankenkasse, TGKK) with the medical examinations of the EVA-Study and health information focusing on CVDs: Potential participants are introduced into the topic of cardiovascular health in a stepwise way. First, adolescents will receive information on the aims of the EVA-project including healthy lifestyle in their school or company (kick-off event). The concept of vascular ageing as a result of continuous accumulation of cardiovascular risks from early life on will be presented orally and by written information material (flyers). The individual visualization of the vessel wall and blood pressure measurement during medical examination will help the adolescents to link this information to their own body. In a second step, individual risk behaviors and laboratory results will be discussed with each participant. In a third step, the summary result from each school/company class will be presented and compared to other schools/companies.

We will create and provide online tools to promote health among adolescents. A website will be designed with information on healthy lifestyle and will contain an encrypted, password-protected web-app that provides the participants with individual health data. Traffic-lights will be used to represent the seven health metrics defined by the AHA [7, 32]. When adding current personal information on body weight, smoking status or physical activity the colors of the traffic lights will switch from red to green (or vice versa) in order to encourage further health-improvements. Further health games (e.g. health quiz) will be added to the website.

Participants will have the opportunity to record their own physical activity over 7 days using modern movement-trackers (Move 3, Movisense GmbH, Karlsruhe, Germany). The results of these records will be downloadable from the web-app.

Special health-promotion programs of the TGKK will include behavioral- and circumstance-oriented activities specially designed for each school/company. Representatives of each school/company are invited for an afternoon workshop aiming at health promotion by oral presentations and written information material. The scholars will undergo an interview to define their interests and expectations. The results of these workshops and the interview will pick out the most relevant topics for a tailored health promotion program for each school or company class. Local health-promotion projects will be supervised by the TGKK and presented on the EVA website as a health promotion book.

### Outcome measures and statistical analysis

The *primary outcome measure* will be the difference in the proportion of each of AHA’s seven health metrics in the ideal range between the intervention and the control group.

The *secondary outcome measure* will be the change in the proportion of AHA’s seven health metrics in the ideal range between baseline and follow-up in the intervention group.

*Further descriptive and exploratory analysis* will focus on the prevalence of unfavorable health behaviors, the prevalence of vascular risk conditions and the change of these factors over age as well as their influence on EVA measured by IMT and the PWV. In addition, the influence of premature birth and early life weight gain (taken from the mother-child-booklet) on obesity and other components of the metabolic syndrome in youth will be explored.

Differences in proportions of the individual health metrics in the ideal range between the intervention and control group (primary outcome parameter) will be analyzed by Chi-squared test. The Prevalence of the individual health metrics in the ideal range in a similar cohort [18] varied from < 5% for ideal diet, over around 50% for ideal physical activity to > 80% for ideal body-mass index. On the basis of a 3:1 intervention to control group split, power analysis indicates that 95% power can be achieved with a sample size of 2000 to detect differences in proportions between intervention and control group of 3% (at a control group proportion of 96%) to 10% (at a control group proportion of 50%) [33] .Multivariate analysis will be done by logistic regression.

### Principal investigators and study-centers

Innsbruck: Univ.-Prof. Dr. Ursula Kiechl-Kohlendorfer (Department of Pediatrics II - Neonatology, Medical University of Innsbruck, Austria), Assoc.-Prof. Priv.-Doz. Dr. Michael Knoflach (Department of Neurology, Medical University of Innsbruck, Austria).

Bruneck: Univ. -Prof. Dr. Ralf Geiger (Azienda Sanitaria dell’Alto Adige, Hospital of Bruneck, Deptartment of Paediatrics and Department of Pediatrics III - Cardiology, Medical University of Innsbruck, Austria).

### Availability of data and materials

Anonymized data can be shared in academic cooperations. Request for data can be addressed to the principal investigators with an appropriate research question.

## Discussion

The EVA-Tyrol study is a large-scale research and prevention program targeting cardiovascular health-promotion in adolescents. The planed 2000 participants will represent about 5% of the Tyrolean population at that age and will allow in-depth insights into vascular risk profile and vascular health of a representative Middle European population. The health promotion program used in our trial could realistically be continued at low costs outside the academic study setting. Our study explores novel grounds, as, even though numerous health promotion programs are targeted to the youth, few did prove efficacy. The STRIP study conducted in Turku, Finland, was able to increase the number of ideal cardiovascular health metrics by accompanying children with dietary and later smoking prevention counselling continuously between the age of 7 month and 20 years. Also, dietary counseling of pre-pubertal children with elevated LDL Cholesterol and their parents effectively reduced LDL Cholesterol [34]. However, other health promotion programs for adolescents were less successful [32, 35]. A recent meta-analysis of 30 studies aiming at improving physical activity in children and adolescents could demonstrate only a modest effect of various interventions [35]. In contrast to our study all these interventions focused on a single health behavior and did not provide comprehensive health counseling on multiple cardiovascular risk factors and behaviors.

The descriptive analysis of the EVA-Tyrol study will substantially enhance our understanding of the distribution of vascular risk behaviors and risk factors in subjects with varying social backgrounds (school types, apprentices) as well as sex and age groups and will help to better focus future health promotion programs.

Furthermore, the EVA-Tyrol cohort will allow to explore mechanisms of early vascular ageing by using well-defined surrogate parameters for vascular health (IMT and PWV) and analyze their association with cardiovascular risk factors and health behaviors. High-quality information from the “mother-child booklet” will elucidate the impact of early life weight gain on adolescent vascular health.

## Data Availability

Request for data shall be addressed to the corresponding authors.

## Abbreviations

AHA: American heart association
COMET: Competence centers for excellent technologies
CRF: Case report form
ctc: Clinical trial center
CVD: Cardiovascular diseases
CVRF: Cardiovascular risk-factors
Dash: Dietary approach to stop hypertension
ECLIA: Electrochemiluminescenceimmunoassay
EVA: Early vascular ageing
FFG: Austrian Research Promotion Agency
HPLC: High pressure liquid chromatography
IHS: International headache society
IMT: Intima-media thickness
MD: Medical doctor
NCD: Non-communicable diseases
PI: Principle investigator
PWV: Pulse wave velocity
TGKK: Tiroler Gebietskrankenkasse = Tyrolean regional medical insurance
VASCage: Research Center of Excellence in vascular ageing - Tyrol
